# Foramen ovale morphology and relationship with the lateral pterygoid process plate: proposal for a new classification system

**DOI:** 10.1007/s12565-025-00826-5

**Published:** 2025-03-01

**Authors:** George Triantafyllou, Panagiotis Papadopoulos-Manolarakis, Sabino Luzzi, Łukasz Olewnik, George Tsakotos, Nicol Zielinska, Renato Galzio, Rǎzvan Costin Tudose, Mugurel Constantin Rusu, Maria Piagkou

**Affiliations:** 1 Department of Anatomy, School of Medicine, Faculty of Health Sciences, National and Kapodistrian University of Athens, 75 Mikras Asias Str., Goudi, 11527 Athens, Greece; 2 Department of Neurosurgery, General Hospital of Nikaia-Piraeus, Athens, Greece; 3 Department of Clinical-Surgical, Diagnostic and Pediatric Sciences, University of Pavia, Pavia, Italy; 4 Department of Clinical Anatomy, Masovian Academy in Płock, Płock, Poland; 5 Division of Anatomy, Faculty of Dentistry, “Carol Davila” University of Medicine and Pharmacy, Bucharest, Romania

**Keywords:** Foramen ovale, Morphology, Classification, Pterygoid process, Clinical anatomy, Trigeminal neuralgia, Percutaneous approach

## Abstract

Surgeons frequently approach the foramen ovale (FO) via the infratemporal fossa (ITF) to treat trigeminal neuralgia. However, this percutaneous procedure could be unsuccessful due to anatomical factors. The present study aimed to assess the FO morphology and its relationship with the lateral pterygoid process plate (LPPP), emphasizing coexisting ITF variants. One-hundred-and-eight (216 sides) adult dried skulls were evaluated at the ITF (FO and LPPP). The FO maximum anteroposterior and lateromedial distances (APD and LMD) were calculated and correlated with the FO morphology. The FO-LPPP relationship was observed, and the presence of a sphenoidal emissary foramen (SEF) and possible ossified sphenoid bone’s ligaments variants (pterygoalar and pterygospinous—PTA and PTS variable ossification) were recorded. Statistical analysis was performed using the SPSS statistical program. The FO morphology was classified into three types after taking into consideration the FO morphometry. Type 1 FO was considered when the APD was two times more than the LMD (45.83%), Type 2 FO was identified when the APD was more than the LMD but no more than two times (51.85%), and Type 3 FO was considered when the APD and LMD were equal (2.32%). The FO and LPPP relationships were classified into the following four types: the direct type when the LPPP base ended at the FO center (32.3%), the lateral type was observed when the LPPP base ended at the FO lateral margin (28.76%), the far type was considered when the LPPP base ended distally to the FO (22.57%), and the medial type was when the LPPP base ended at the FO medial margin (11.95% of cases). Concerning the impact of the sphenoid bone variants on the FO-LPPP, the SEF did not alter the FO-LPPP relationship, while the PTA or PTS bar presence significantly affected it (*p* < 0.001 and *p* = 0.007, respectively). When the sphenoid bone ossified bars were present, the most common type of FO was the medial one. A novel classification system was proposed for the FO morphology, assessing possible coexisting ITF variants that could alter the FO-LPPP relationship. Knowledge of these details would help clinicians perform percutaneous procedures to treat trigeminal neuralgia.

## Introduction

The skull base depicts exciting typical anatomy and variants essential for anatomists, radiologists, and intervening neurosurgeons. Several procedures, such as tumor resection, are performed in the intracranial and extracranial skull base. The high morphological variability of these regions can implicate these procedures. (Antonopoulou et al. 2008; Henry et al. 2020; Iwanaga et al. 2022; Li et al. 2021; Natsis et al. 2018, 2014; Pękala et al. 2017; Piagkou et al. 2023a, b; Prakash et al. 2019; Rusu 2024; Šink et al. 2023; Tubbs et al. 2009; Abdelghani et al. 2024).

The sphenoid bone greater wing has several foramina at its medial aspect, including the foramina ovale and spinosum (FO and FS). The FO has a medial position to the FS and a lateral position to the foramen lacerum (FL) on the infratemporal surface. The trigeminal nerve mandibular division (V3) courses through the FO, along with the maxillary artery accessory meningeal branch and an emissary vein connecting the cavernous sinus with the pterygoid venous plexus (Standring et al. 2016). The FO emissary veins have been described as venous channels laterally to the V3 (Leonel et al. 2018; Tsutsumi et al. 2020). Multiple variants have been described in this area, such as an accessory foramen, the so-called sphenoidal emissary foramen (SEF), or “foramen Vesalius” (Leonel et al. 2020; Piagkou et al. 2023b), as well as the sphenoid bone’s ossified ligaments (pterygoalar and pterygospinous ligaments—PTA and PTS), which may be partially or wholly ossified, thus creating bony arches (Pękala et al. 2017; Henry et al. 2020; Matys et al. 2020). These well-studied variants can significantly complicate the infratemporal fossa anatomy.

Clinically, the FO is reached percutaneously as a treatment option for trigeminal neuralgia patients (Iwanaga et al. 2022). Therefore, Iwanaga et al. (2022) studied the anatomical relationship between the FO and the lateral pterygoid process plate (LPPP). The classification system for the FO-LPPP relationship was as follows:

- Type I corresponded to the base of the LPPP posterior border reaching the FO lateral margin—“lateral type”.
- Type II corresponded to the LPPP base posterior border reaching the FO medial margin—“medial type”.
- Type III corresponded to the LPPP base posterior border ending at or close to the FO center—“direct type”.
- Type IV corresponded to the LPPP base posterior border, which ended distant from the FO—“removed type”.

Herein, we performed a gross anatomy study investigating the FO morphology and its relationship with the LPPP according to Iwanaga et al. (2022). We aimed to identify if coexisting variants, such as the presence of the SEF and the ossification of the sphenoid bone’s extracranial ligaments, affected this relationship (FO-LPPP).

## Materials and methods

One hundred and twenty (120) adult dried skulls were screened for eligibility for the study. The exact ages were unknown, and the sexes were distributed as 28 males, 25 females, and the rest were of unknown sex. Excluding criteria were injuries on the FO and/or LPPP and/or FO and FS confluence. The sample belongs to the skeletal collection of the Department of Anatomy (Medical School, National and Kapodistrian University of Athens), derived from the Body Donation Program (Brenner et al. 2024). The authors hereby confirm that every effort was made to comply with all local and international ethical guidelines and laws concerning the use of human cadaveric donors in anatomical research.

We measured the FO maximum anteroposterior and lateromedial diameters (APD and LMD). The horizontal relationship between the FO and LPPP posterior border was bilaterally observed from an extracranial view of the skull base, as suggested by Iwanaga et al. (2022). Additionally, we observed coexisting variants: 1) the SEF presence and 2) the ossification pattern of the sphenoid bone’s extracranial ligaments (PTS and PTA).

Statistical analysis was performed with IBM Statistics for MacOS IBM SPSS Statistics for MacOS, Version 29 (IBM Corp., Armonk, New York, United States). Nominal data (FO types, FO-LPPP types, SEF presence, PTA/PTS presence) between sexes and types were compared using the Chi-square test, while McNemar’s test was applied for sides. Normality was assessed with the Shapiro–Wilk test. Continuous variables (FO APD and LMD measurements) were analyzed based on measurement type: unpaired measurements were evaluated with an independent t-test if normality was met (FO APD measurement); otherwise, the Mann–Whitney U test was used (FO LMD measurement). The results are presented as mean and standard deviation unless otherwise specified. A *p* value less than 0.05 was considered statistically significant.

## Results

One-hundred-and-eight dried skulls (216 sides) were eligible for the current study.

### Foramen ovale (FO) morphology and morphometry

**Fig. 1:**
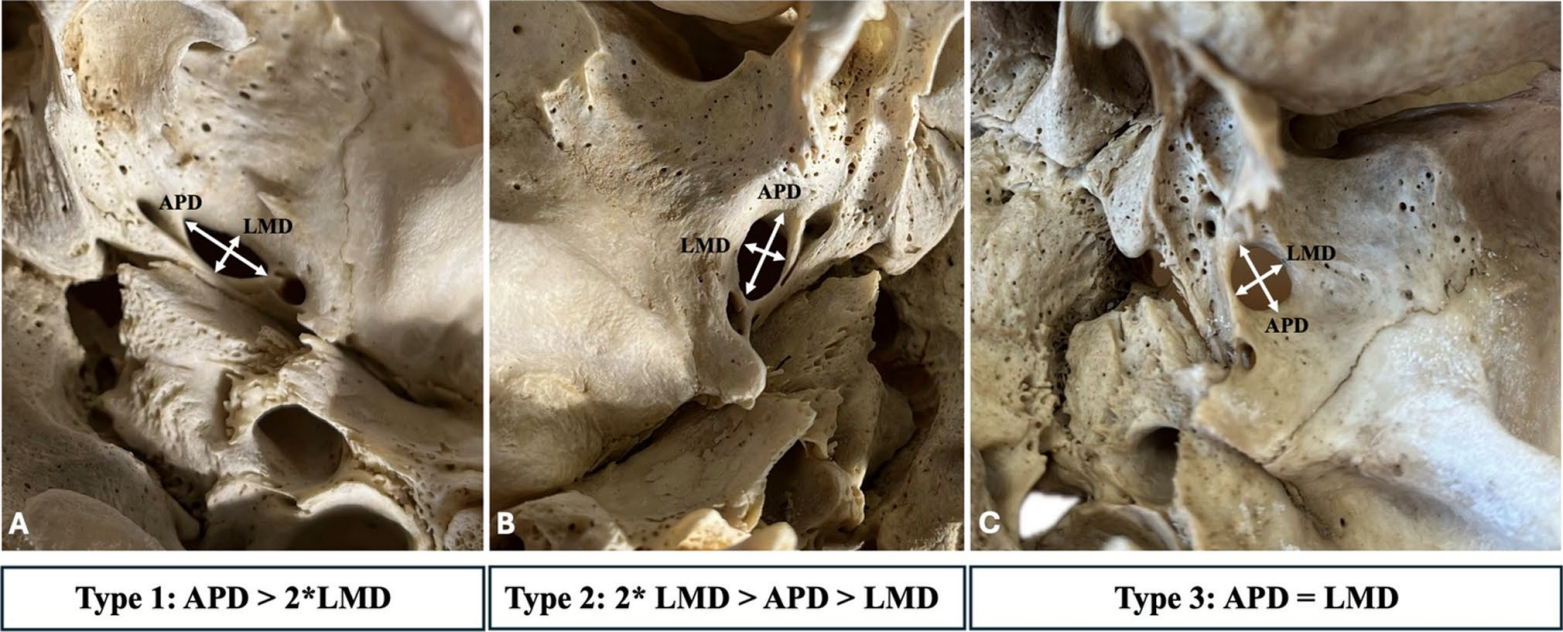
The proposed classification system for the foramen ovale (FO) morphology, after considering the foramen’s morphometry. *Maximum APD* maximum anteroposterior distance, *maximum LMD* maximum lateromedial distance

The FO morphometry and morphological variability (classified in types) are summarized in Tables 1 and 2. The FO maximum APD was 8.71 ± 1.58 mm with no significant difference between the sides (*p* = 0.071) and sexes (*p* = 0.123). The FO maximum LMD was 4.71 ± 1.27 mm with no significant association between sides (*p* = 0.878) and sexes (*p* = 0.701) (Table 1). These measurements were used to classify the FO morphology. Type 1 was considered when the APD was twice more than the LMD (APD > 2 × LMD). Type 2 when the APD was more than the LMD but less than twice (2 × LMD > APD > LMD). Type 3 was characterized when the APD was equal to the LMD (LMD = APD) (Fig. 1). No significant association between sides (*p* = 0.766) and sexes (*p* = 0.067) was identified for the different FO types (Table 2). The laterality of the FO morphology is presented in Table 3. The most common type was the combination of Types 1 and 2 (37.96%). The symmetrical FO morphology (same type bilaterally) was identified in 56.48%, while the asymmetrical morphology (different type bilaterally) was recorded in 43.52%.

**Table 1:** Foramen ovale (FO) morphometry overall and according to sides and sexes

FO morphometry	Total *N* = 216	Left *n* = 108	Right *n* = 108	*p* value	Males *n* = 56	Females *n* = 50	*p* value
APD (mm)	8.71 (1.58)	8.51 (1.54)	8.90 (1.60)	0.071	8.99 (1.59)	8.50 (1.58)	0.123
LMD (mm)	4.71 (1.27)	4.70 (1.33)	4.71 (1.22)	0.878	4.75 (1.35)	4.64 (1.35)	0.701

The results are presented as mean (standard deviation)

*mm* millimeters, *APD* anteroposterior distance, *LMD* lateromedial distance

**Table 2:** Foramen ovale (FO) morphology overall and according to sides and sexes

FO morphology	Total *N* = 216 (%)	Left *n* = 108 (%)	Right *n* = 108 (%)	*p* value	Males *n* = 56 (%)	Females *n* = 50 (%)	*p* value
Type 1	99 (45.83)	42 (38.89)	57 (52.78)	0.067	27 (48.21)	24 (48)	0.766
Type 2	112 (51.85)	65 (60.19)	47 (43.52)		27 (48.21)	25 (50)	
Type 3	5 (2.32)	1 (0.93)	4 (3.70)		1 (1.78)	1 (2)	

Type 1—APD > 2 × LMD, Type 2—2 × LMD > APD > LMD, Type 3—APD = LMD

**Table 3:** Combinations of foramen ovale (FO) morphology bilaterally

Type 1–1 *n* (%)	Type 2–2 *n* (%)	Type 3–3 *n* (%)	Type 1–2 *n* (%)	Type 1–3 *n* (%)	Type 2–3 *n* (%)
26 (24.07)	35 (32.41)	0 (0)	41 (37.96)	0 (0)	6 (5.56)

Type 1—APD > 2 × LMD, Type 2—2 × LMD > APD > LMD, Type 3—APD = LMD

### Foramen ovale (FO) relationship with lateral pterygoid process plate (LPPP)

**Fig. 2:**
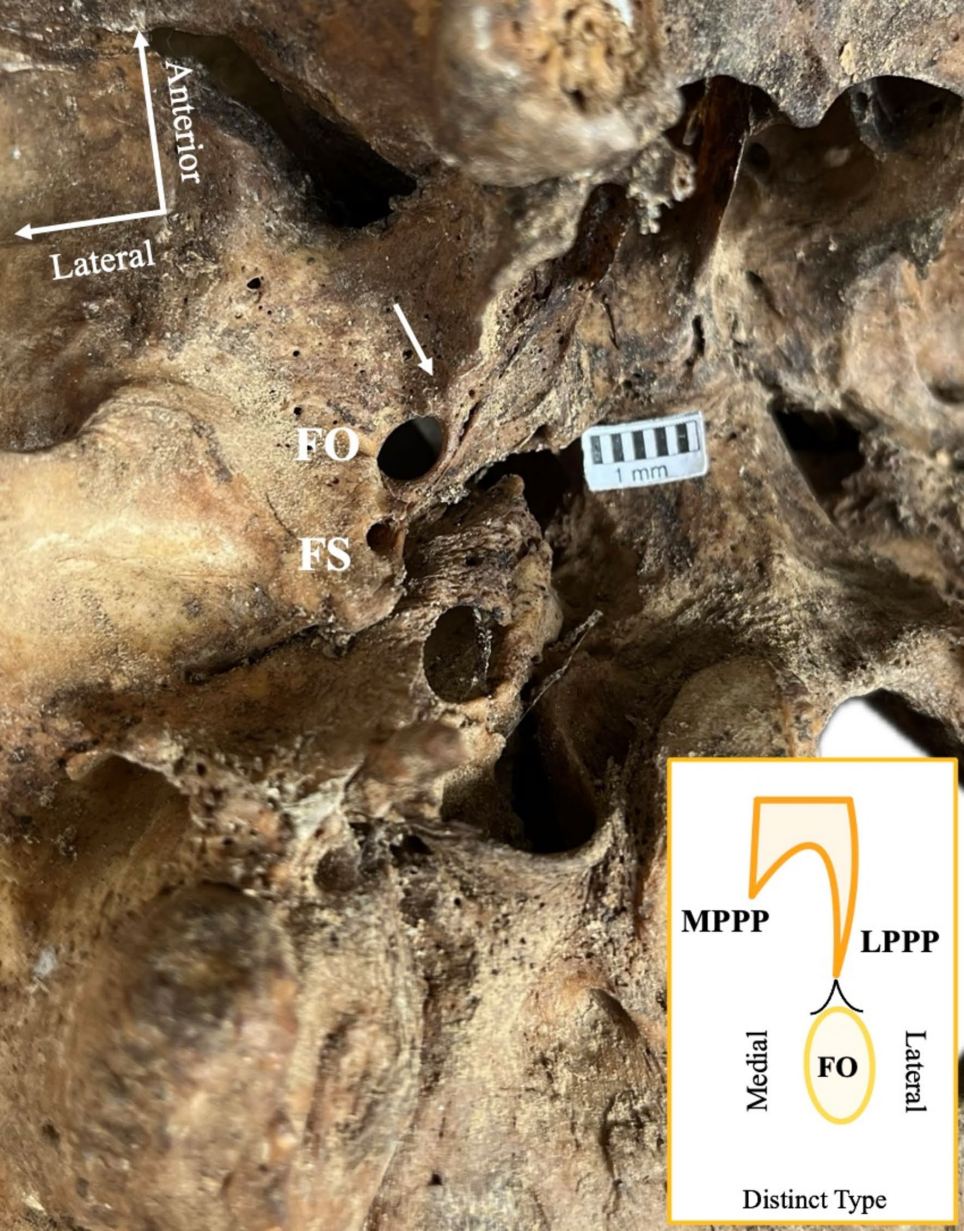
The “direct type” relationship (arrow) between the foramen ovale (FO) and the lateral pterygoid process plate (LPPP). *FS* foramen spinosum

**Fig. 3:**
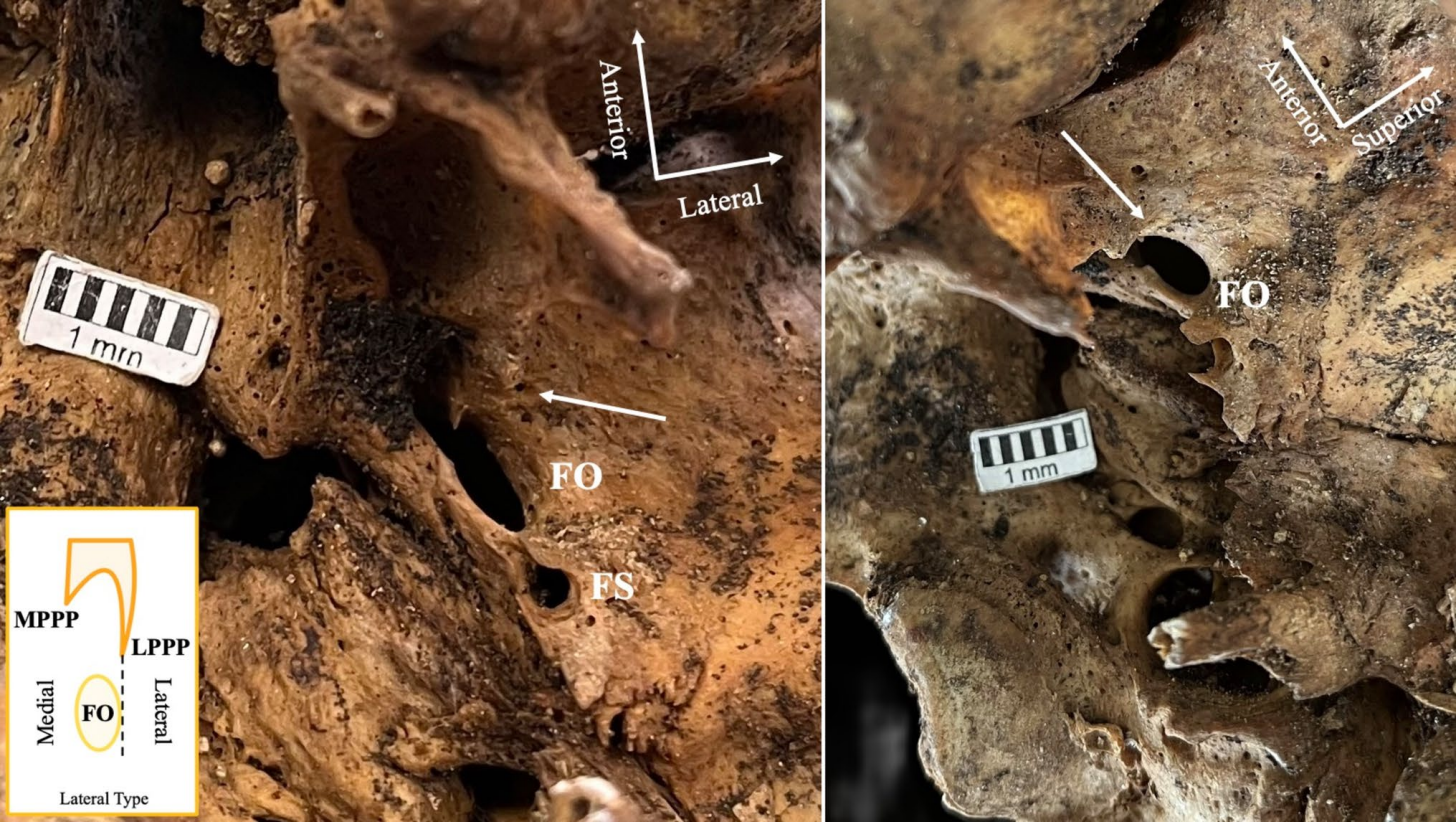
The “lateral type” relationship (arrow) between the foramen ovale (FO) and the lateral pterygoid process plate (LPPP). *FS* foramen spinosum

**Fig. 4:**
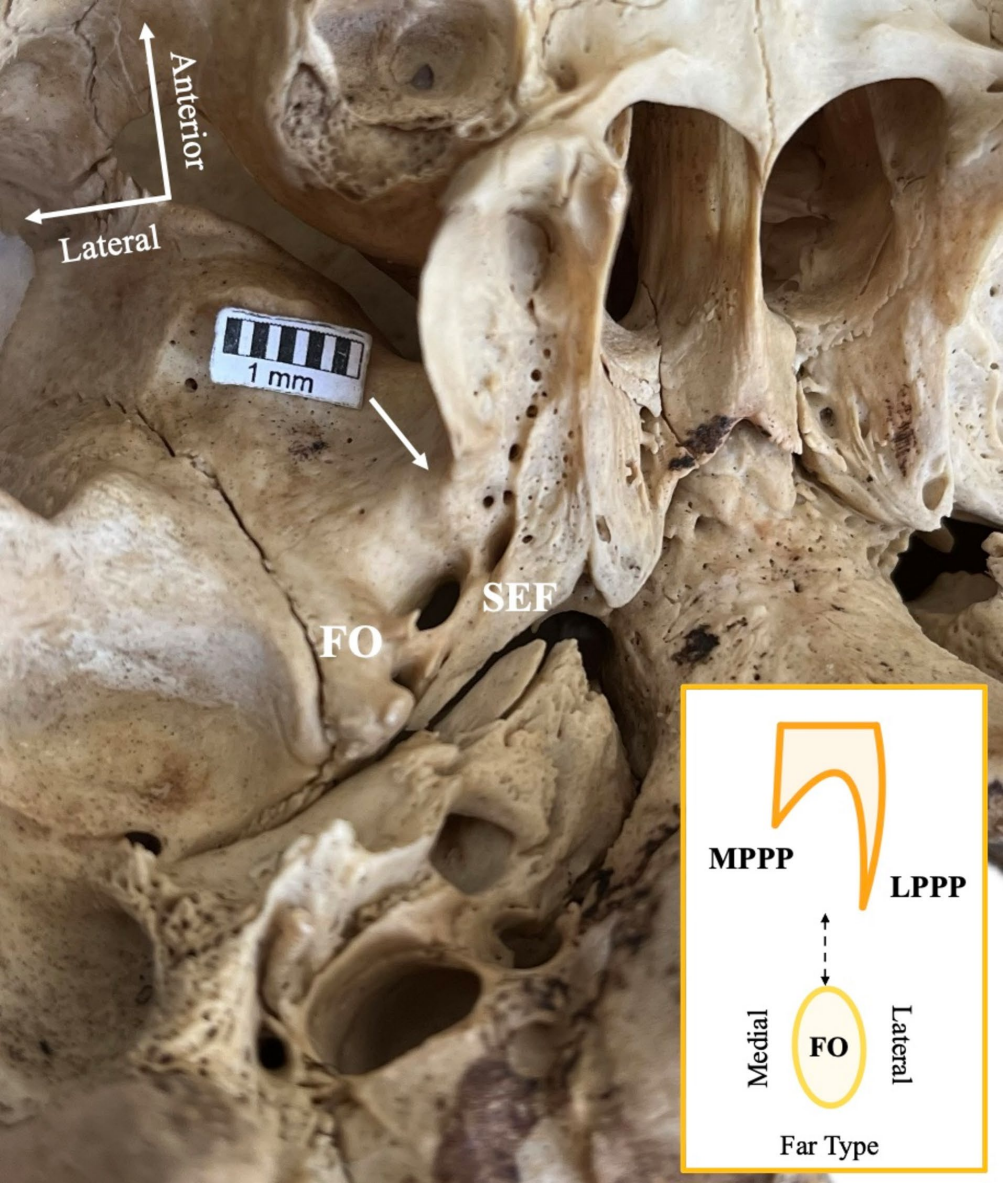
The “far type” relationship (arrow) between the foramen ovale (FO) and the lateral pterygoid process plate (LPPP). *SEF* sphenoidal emissary foramen

**Fig. 5:**
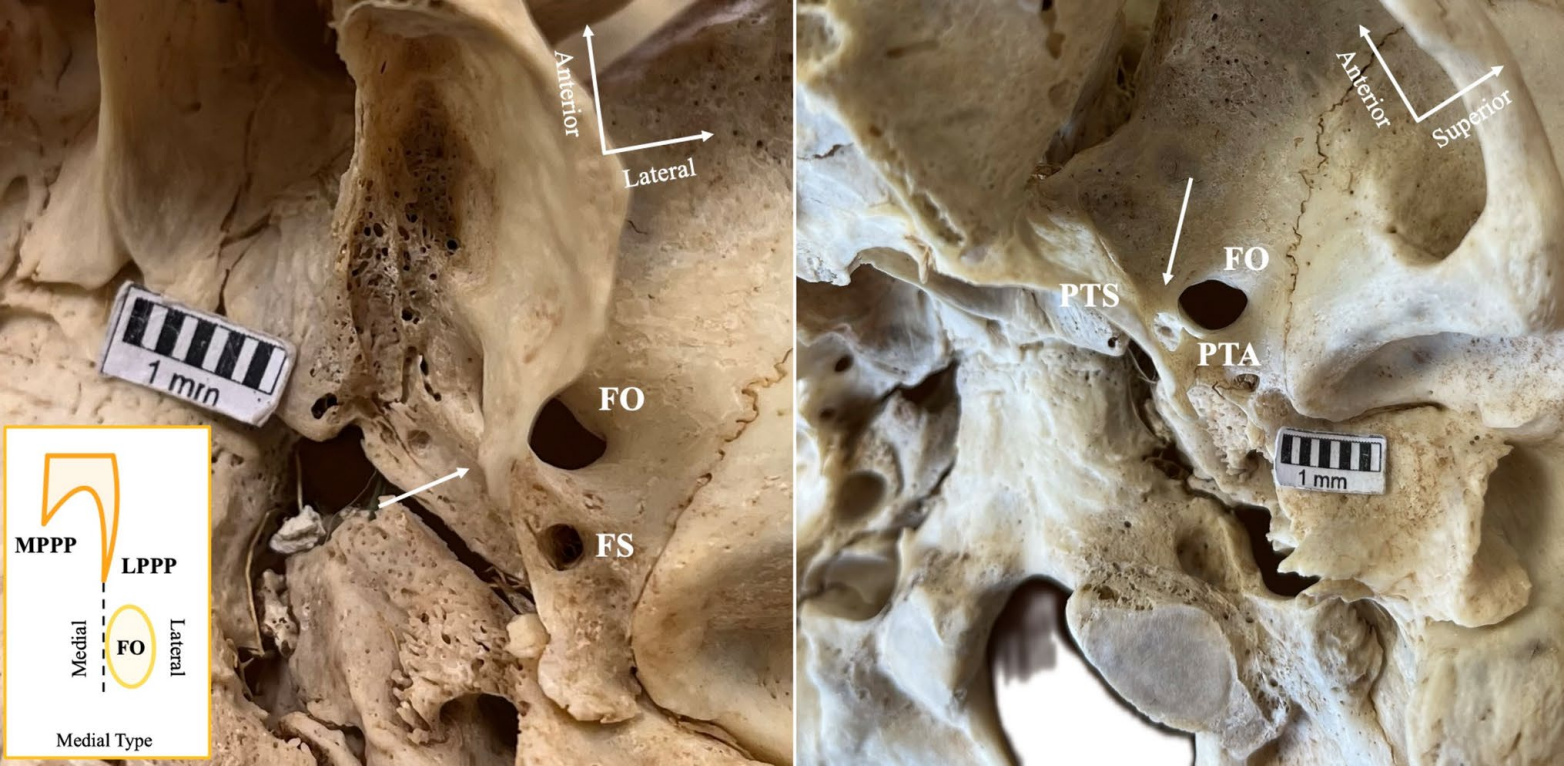
The “medial type” relationship (arrow) between the foramen ovale (FO) and the lateral pterygoid process plate (LPPP). Note that this skull corresponds to the coexistence of pterygoalar (PTA) and pterygospinous (PTS) bars. *FS* foramen spinosum

The FO-LPPP relationship results are summarized in Table 4. The most common type was the direct type, identified when the base of the LPPP ended at the FO center (32.3%) (Fig. 2). The lateral type was characterized when the base of the LPPP ended at the FO lateral margin (28.76%) (Fig. 3). The *far type* was observed when the base of the LPPP ended far from the FO (22.57%) (Fig. 4). The rarest type corresponded to the cases in which the base of the LPPP ended at the FO medial margin (11.95%) (Fig. 5). No association was identified between the sides and sexes (Table 4). The laterality of the FO-LPPP topographical relationship is summarized in Table 5. A symmetric relationship (same type bilaterally) was identified in 36.11%.

**Table 4:** Foramen ovale (FO) relationship with the lateral pterygoid process plate (LPPP) overall and according to sides and sexes

FO-LPPP relationship	Total *N* = 216 (%)	Left *n* = 108 (%)	Right *n* = 108 (%)	*p* value	Males *n* = 56 (%)	Females *n* = 50 (%)	*p* value
Direct	73 (33.80)	37 (34.26)	36 (33.33)	0.878	23 (41.07)	15 (30)	0.277
Lateral	65 (30.09)	34 (31.48)	31 (28.70)		11 (19.64)	17 (34)	
Removed	51 (23.61)	23 (21.30)	28 (25.93)		15 (26.79)	15 (30)	
Medial	27 (12.5)	14 (12.96)	13 (12.04)		7 (12.5)	3 (6)

**Table 5:** Combinations of foramen ovale (FO) relationship with the lateral pterygoid process plate (LPPP) bilaterally

Type 1–1 *n* (%)	Type 2–2 *n* (%)	Type 3–3 *n* (%)	Type 4–4 *n* (%)	Type 1–2 *n* (%)	Type 1–3 *n* (%)	Type 1–4 *n* (%)	Type 2–3 *n* (%)	Type 2–4 *n* (%)	Type 3–4 *n* (%)
16 (14.81%)	6 (5.56%)	9 (8.33%)	8 (7.41%)	2 (1.85%)	23 (21.30%)	10 (9.26%)	11 (10.19%)	2 (1.85%)	21 (19.44%)

*Type 1* lateral type, *Type 2* medial type, *Type 3* direct type, *Type 4* removed type

### Coexisted variations in the infratemporal fossa (ITF)

**Fig. 6:**
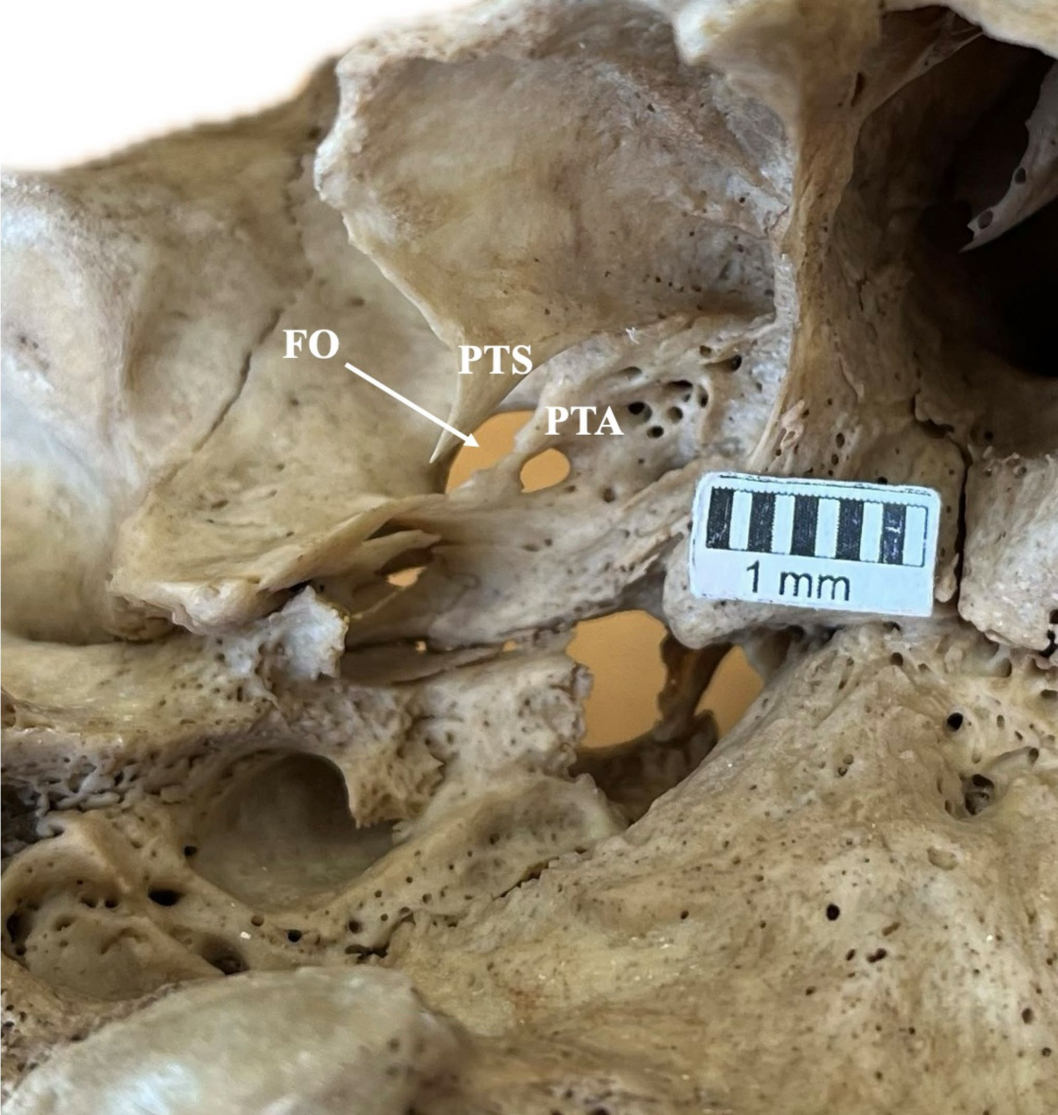
Coexistence of pterygoalar (PTA) and pterygospinous (PTS) bars. Note that the foramen ovale (FO) and lateral pterygoid process plate (LPPP) relationship is the “medial type”

The presence of coexisted infratemporal fossa variants is summarized in Table 6 (Figs. 5, 6).

**Table 6:** Foramen ovale (FO) relationship with the lateral pterygoid process (LPP) when the sphenoidal emissary foramina (SEF) or pterygoalar bar (PTA) was present

Parameters	Absent SEF *n* = 171 (%)	Present SEF * n* = 45 (%)	*p* value	Absent PTA * n* = 203 (%)	Present PTA * n* = 13 (%)	*p* value	Absent PTS * n* = 204 (%)	Present PTS * n* = 12 (%)	*p* value
Direct type	61 (35.67)	12 (26.67)	0.414	69 (33.99)	4 (30.77)	< 0.001*	69 (33.82)	4 (33.33)	0.007*
Lateral type	53 (30.99)	12 (26.67)		64 (31.53)	1 (7.69)		64 (31.37)	1 (8.33)	
Removed type	37 (21.64)	14 (31.11)		51 (25.12)	0 (0)		50 (24.51)	1 (8.33)	
Medial type	20 (11.70)	7 (15.56)		19 (9.36)	8 (61.54)		21 (10.29)	6 (50)	

The asterisk highlights the significant result

The SEF was identified in 45 sides (20.83%), bilaterally in 16 skulls, and unilaterally in 13 skulls. No significant difference between sides (*p* = 0.867) and sexes (p = 0.689) was identified. The SEF did not influence the FO-LPPP relationship (*p* = 0.414). The FO APD was measured at 8.33 ± 1.62 mm when the SEF was present, while it was measured at 8.81 ± 1.56 mm when the SEF was absent (*p* = 0.072). The FO LMD was 4.47 ± 1.29 mm when the SEF was present, while it was 4.77 ± 1.27 mm when the SEF was absent (*p* = 0.068).

The PTA bar was identified on 13 sides (6.02%), unilaterally in nine skulls and bilaterally in three skulls. No significant association existed between the sides (*p* = 0.874) and sexes (*p* = 0.391). The FO-LPPP relationship was substantially different between the PTA bar absence and presence (*p* < 0.001). When the PTA bar was present, the FO-LPPP relationship was the medial type, most commonly (61.54% of the skulls).

The PTS bar was identified in 12 sides (5.56%), unilaterally in 8 skulls and bilaterally in 2 skulls. A left-sided predominance (*p* = 0.017) and no significant association between sexes (*p* = 0.101) was identified. The FO-LPPP relationship was substantially different between the PTS bar absence and presence (*p* = 0.007). In cases of PTS bar presence, the FO-LPPP relationship was the medial type, most commonly (50% of the skulls).

The PTA and PTS bars’ coexistence was identified in 5 skulls (Figs. 5, 6). In these skulls, the FO-LPPP relationship was the medial type (in 3 skulls) and the direct type (in 2 skulls).

## Discussion

In the present investigation, we evaluated the FO morphology and its relationship with the LPPP. We have introduced an innovative classification system for FO morphology by considering the morphometric details and we are examining concurrent variants that may impact the correlation with the LPPP. We identified that the PTA and PTS bars significantly influenced the FO-LPPP relationship.

### Foramen ovale (FO) morphology and morphometry

The FO morphology has been well studied. Prakash et al. (2019) studied the FO in 62 dried skulls (124 sides) and classified it into four types: the typical oval shape (60.4%), the almond shape (28.22%), the round shape (6.45%), and the irregular (3.22%). Elnashar et al. (2020) studied the FO in 174 dried skulls to enhance the understanding during percutaneous rhizotomy. They identified six possible FO shapes: oval (38.2%), crescent (31.2%), cordate (12%), almond (9.4%), elongated (7.2%), and round (2.9%). Although both studies proposed a classification system based on their findings, the FO morphological identification using different shapes is inaccurate. The same problem arose from other human body regions, such as the suprascapular notch (Al-Redouan et al. 2021; Tsakotos et al. 2024), the superior orbital fissure (Regoli and Bertelli 2017), and the spina recti lateralis (Bonente et al. 2024). Different morphological shapes were proposed for the suprascapular notch classification; however, the most recent studies suggested that the most accurate way is to classify the suprascapular notch according to its morphometric details (Al-Redouan et al. 2021; Tsakotos et al. 2024). Therefore, we used a similar method to classify the FO with the most accurate method. This novel classification system using the FO morphometrical details is scientifically more accurate than characterizing shapes that could lead to pitfalls due to their subjectivity.

### Relationship between foramen ovale (FO) and lateral pterygoid process plate (LPPP)

A unique classification system for the FO-LPPP relationship was proposed by Iwanaga et al. (2022), as follows:

- Type I “lateral type” was identified in 29% of the study and 30.09% of our research.
- Type II “medial type” was observed in 15% of Iwanaga et al. (2022) study and 12.5% of our research.
- Type III “direct type” was identified in 35% of Iwanaga et al. (2022) study and 33.80% of our research.
- Type IV “removed/far type” was observed in 21% of Iwanaga et al. (2022) study and 23.61% of our research.

The results of the two studies were quite similar. Iwanaga et al. (2022) noted a left-sided predominance in the “medial type,” which we did not observe in the current study (Table 4). Additionally, we present the combinations of types based on laterality. The most frequent combination was the “lateral type” with the “direct type” in 21.30% of skulls (Table 5).

### Sphenoidal emissary foramina (SEF) effect

The study also documented the SEF presence, which was found in 20.83% of cases, typically located anteriorly or anteromedially to the FO. The SEF provides a passage for a vein from the pterygoid venous plexus to connect with the cavernous sinus. A systematic review with meta-analysis examined the SEF presence in 6,369 dried skulls, revealing an overall prevalence of 38.1% (Piagkou et al. 2023b). Our prevalence was lower than the current meta-analysis; this could be attributed to the fact that we determined the SEF when it was present in both extracranial and intracranial (with the use of metal wire), while in the current literature, many studies reported the SEF presence only intracranially (Piagkou et al. 2023b).

Our study determined whether the SEF presence affected the FO-LPPP relationship. However, the statistical analysis yielded no significant impact (*p* = 0.414). Another investigation explored whether the SEF’s presence significantly alters the FO’s morphometry (Natsis et al. 2018). Similarly to this study, we did not observe a statistically significant difference.

### Sphenoid bone ligaments’ ossification effect

Excluding the SEF, we noted the potential impact of the PTA and PTS bar on the FO-LPPP relationship. Typically, the PTS bar is more common than the PTA bar. (Pękala et al. 2017; Henry et al. 2020). However, in the current study, the PTA bar was more common than the PTS bar, similar to our previous study (Piagkou et al. 2023a). The PTA bar has been extensively researched using dried skulls or computed tomography scans due to its neurosurgical significance (Antonopoulou et al. 2008; Iwanaga et al. 2020; Natsis et al. 2014; Pękala et al. 2017; Piagkou et al. 2023a; Tubbs et al. 2009). Iwanaga et al. (2020) suggested that the most accurate method is the cadaveric investigation with soft tissues to understand whether the PTA bar corresponds to an ossified PTA ligament. Furthermore, Pękala et al. (2017) conducted a meta-analysis of its pooled prevalence. They identified the incomplete PTA bar prevalence at 8.4% and the complete one at 4.4%. In our study, the PTA bar was observed in 6.02% of cases, regardless of its complete or incomplete type. Our study observed that the FO-LPPP relationship altered when the PTA bar was present. Specifically, the “medial type” was most commonly observed in 61.54% of skulls with the PTA bar.

Similar to the PTA bar, the PTS bar variants have been well described in the current literature. (Antonopoulou et al. 2008; Henry et al. 2020; Iwanaga et al. 2020; Piagkou et al. 2023a). Henry et al. (2020) calculated the pooled prevalence of the complete PTS bar at 4.4% and the incomplete one at 11.6%. Our study identified the PTS bar in 5.56% of cases, regardless of its complete or incomplete type. We determined that the FO-LPPP relationship was affected by the presence of the PTS bar. The “medial type” was most commonly identified in 50% of skulls with the PTS bar.

### Clinical significance

The clinicians who perform a percutaneous approach for trigeminal neuralgia must have detailed knowledge of FO and its surrounding structures. Computed tomography can help the surgeon improve the accuracy and safety of the FO cannulation. However, even with navigation technology, unsuccessful cannulation of the FO has been reported. The use of an intraoperative CT scan obtained using the Medtronic O-arm for image-guided cannulation of the FO during percutaneous rhizotomy procedures, not previously accessible with fluoroscopy alone, was also described. This technique does not require the placement of an invasive head clamp and may be used with an awake patient. Using image guidance, the authors navigated the needle to percutaneously access the FO by using a single tract to successfully complete balloon compression of the trigeminal nerve (Bohnstedt et al. 2012; Héréus et al. 2023).

Elnashar et al. (2020) simulated the cannulation in dried skulls and compared the FO morphology between the gross anatomical and surgical views. Although they used a classification system with shapes that we are depicting as inaccurate, they observed that the FO morphology is significantly different when the FO is observed from the surgical view. They highlighted that sometimes the LPPP obstructed the FO view (Elnashar et al. 2020). Keeping this in mind, Iwanaga et al. (2022) proposed a classification system for the relationship between the FO and LPPP. They categorized it into Iwanaga types I to III (lateral, medial, and direct), with an overall prevalence of 79% (Iwanaga et al. 2022) and 77.5% (current study). These types provide easy access to the FO because the LPPP base ends close to the FO (proximal), and the needle will be led there (Iwanaga et al. 2022). On the other hand, Type IV (removed type) might cause difficulties for clinicians, as the LPPP base is far from the FO (Iwanaga et al. 2022). We also noted that the PTA or PTS bars are possible covariant altering this relationship. The SEF presence did not affect it; however, it has been reported that the SEF could be misdiagnosed as the FO and lead to the failure of FO approaches (Iwanaga et al. 2022). The PTA and PTS bars affected this relationship, and when present, the medial type was statistically more common. It is well known that the PTA or PTS bar corresponds to other causes of failure during percutaneous procedures (Antonopoulou et al. 2008; Iwanaga et al. 2020; Natsis et al. 2014; Piagkou et al. 2023a; Tubbs et al. 2009). Although the “medial type” is an easy-to-access relationship, the PTA or PTS bar coexistence could precipitate difficulties; for example, the needle might be inserted into the foramen formatted by the PTA or PTS bar instead of the FO. Nevertheless, we identified five sides (2.31% of our sample) with both PTS and PTA bars. Although this could be considered infrequent, the coexistence of these bars might precipitate extreme difficulties for the surgeon trying to access the FO.

Entrapment neuropathies are compressive neuropathies that occur when nerves are confined within narrow anatomical passageways formed by soft and hard tissues, making them vulnerable to constricting pressure. Chronic nerve compression can disrupt the nerve's normal anatomical and functional integrity. Various anatomical structures can contribute to the entrapment and compression of the posterior trunk of the mandibular nerve. The FO width is a significant factor affecting the mandibular nerve and the surrounding structures' topographic location (Natsis et al. 2015; Piagkou et al. 2011a, b). Moreover, the ossified PTA ligament can compress the buccal, lingual, and auriculotemporal nerves, leading to symptoms such as numbness, paresthesia, hypoesthesia, anesthesia, and trigeminal neuralgia (Piagkou et al. 2010; Natsis et al. 2015). Additionally, the ossification of the PTS may result in trigeminal neuralgia through the entrapment of the mandibular branches (Antonopoulou et al. 2008; Piagkou et al. 2011a, b). Furthermore, these ossified ligaments can affect the blood vessels supplying the trigeminal ganglion, which may damage the mandibular nerve (Piagkou et al. 2011a, b).

### Limitations

There are certain limitations associated with the current study. The study sample (*n* = 120 skulls) was considered adequate for the FO morphology and FO-LPPP relationship; however, a higher sample would have been desirable for the PTA and PTS bars identification, as well as the gender and age impact. Moreover, by using dry skulls with no soft tissue, the exact identification of PTA and PTS bars according to Iwanaga et al. (2020) was impossible.

## Conclusions

The study re-evaluated the FO morphology using morphometric parameters and introduced a precise and innovative classification system. The study proposed three types based on the FO's maximum APD and LMD. Additionally, the study examined the relationship between the FO and the LPPP and investigated the impact of coexisting variants. The presence of the PTA and PTS bars significantly affected this relationship. The FO morphology and its relationship with the LPPP are crucial for neurosurgeons treating trigeminal neuralgia to ensure successful outcomes and prevent complications.

## Data Availability

All the data are available upon reasonable request to the corresponding author (George Triantafyllou- email: georgerose406@gmail.com).
